# Increasing wheat flour extraction rate for balanced starch-protein digestion and gut microbiota optimization: A strategy to enhance nutrition and mitigate food crises

**DOI:** 10.1016/j.fochx.2025.102848

**Published:** 2025-07-30

**Authors:** Lingfang Zhang, Peng Chen, Haowen Lu, Qianyi Jiang, Fan Yang, Binghua Sun, Xiaoxi Wang

**Affiliations:** College of Food Science and Engineering, Henan University of Technology, Zhengzhou, Henan Province, PR China

**Keywords:** Wheat flour extraction rate, Starch hydrolysis degree, Protein hydrolysis degree, *In vitro* fermentation

## Abstract

In this study, the influence of wheat flour extraction rate on starch and protein hydrolysis degree, as well as fermentation characteristics of noodles was evaluated using an *in vitro* digestion-fermentation model. Results showed that as the flour extraction rate increased from 35 % to 95 %, the starch hydrolysis degree of noodles significantly decreased. Meanwhile, the protein hydrolysis degree (*n* = 3) exhibited a higher value of 22.56 %–23.53 % in noodles with an 80 %–90 % extraction rate, then declined to 21.69 % at a 95 % extraction rate. Noodles with an extraction rate of 75 % to 85 % produced significantly higher levels of short-chain fatty acids (247.19–254.73 mmol/L, *n* = 6) than other samples. Moreover, the ratio of Bacteroidetes/Firmicutes also increased with flour extraction rate, positively regulating gut microbiota. Notably, noodles with an 80 %–85 % extraction rate could maintain intestinal microecological balance. Overall, increasing flour extraction rate to 80 %–85 % balanced reduced starch digestibility, elevated protein utilization, and improved gut microbiota stability.

## Highlights

• 85 % wheat flour extraction rate noodles enhanced protein bioaccessibility.
• Noodles with flour extraction rates ≥80 % enrich beneficial gut bacteria.
• Noodles with 95 % flour extraction rate targeted *Bifidobacterium* proliferation.

## Graphical abstract

**Figure:**
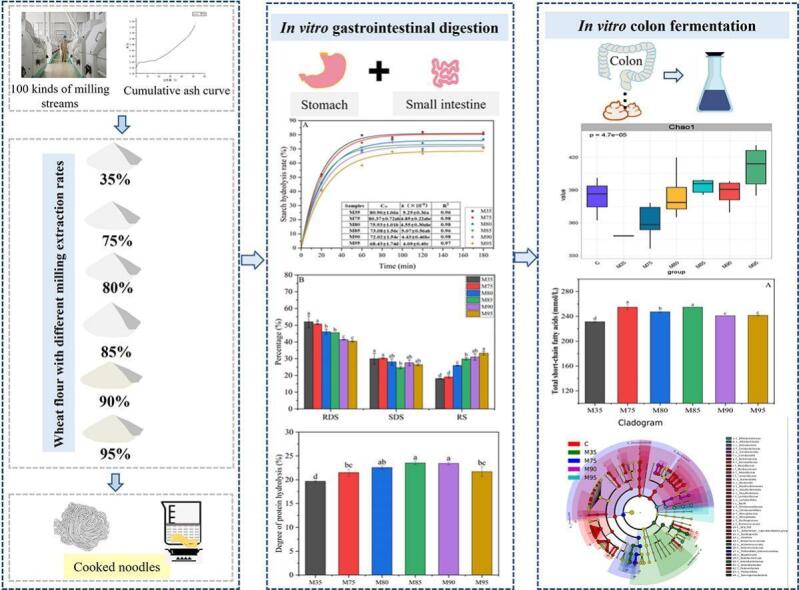

## 1 Introduction

Wheat flour, a globally significant ingredient of staple foods, serves as a strategic resource to fulfill fundamental energy and protein nutritional demands in economically disadvantaged regions. According to the Food and Agriculture Organization (FAO), wheat flour contributes the most abundant plant protein source (20 %) and caloric supply (30 %) to human diets (Cetiner & Koksel, 2024).

Wheat flour extraction rate (also referred to as milling extraction rate), which is closely associated with the processing quality and nutritional quality of wheat flour, refers to the mass fraction of the finished wheat flour relative to the total mass of whole wheat kernels. During industrial milling, refined flour is often produced by removing wheat bran and germ, with extraction rates typically below 75 %, resulting in significant nutrient loss (Zhang, Yang, et al., 2024). Comparative analyses have demonstrated that reducing the extraction rate from 95 % to 61.5 % leads to a 14 % loss of protein (primarily albumin and globulin), and a 29 % loss of dietary fiber in wheat flour (Yılmaz et al., 2018). This nutrient loss during wheat milling contrasts sharply with the stark realities outlined in the Global Report on Food Crises (GRFC) 2024, which highlights that 281.6 million individuals worldwide experienced an acute food crisis in 2023 (Network & Crises, 2024). Therefore, reevaluating the wheat milling process is necessary, as it represents a key breakthrough in alleviating the global food crisis. Increasing the extraction rate of wheat flour (>75 %) provides a solution to reduce waste while preserving higher contents of nutrients, particularly protein, during industrial milling. (Zhang, Yang, et al., 2024) reported that the proportion of total protein, albumin, globulin, and wheat bran dietary fiber in wheat flour increased significantly with flour extraction rate. However, the mere retention of more nutrients in wheat flour does not inherently translate to higher nutritional value. The nutritional value of wheat flour is also determined by the digestibility and fermentability of its nutrients (Fang et al., 2025). Existing research has remained confined to superficial analyses of the nutritional composition of wheat flour with varying flour extraction rates. These studies failed to sufficiently address how variations in wheat flour extraction rate influence the digestibility of its nutritional composition, particularly through alterations in endogenous components such as wheat bran dietary fiber. Previous studies found that elevated dietary fiber content in food markedly decreased protein digestibility through two mechanisms: (1) steric hindrance impeding protease accessibility, and (2) competition for water binding capacity limiting protein hydration (Wen et al., 2020; Zhang, Qian, et al., 2024). Concurrently, dietary fiber also reduces the digestibility of starch by inhibiting amylase activity (Qi et al., 2016). Notably, proteins, starch, and dietary fiber in wheat flour interact synergistically—*e.g.*, *via* protein-fiber binding or starch entrapment in fiber matrices—modulating their digestive processes. Importantly, the manner in which this synergistic effect is regulated by varying extraction rates remains underexplored in current literature. As a result, it is reasonable to speculate that changes in dietary fiber content caused by increased flour extraction rate may regulate the digestibility of protein and starch in wheat flour.

Moreover, changes in endogenous components caused by wheat flour extraction rate not only affect nutrient digestibility but also influence subsequent colonic fermentation characteristics. Protein digestibility in the small intestine determines the amount of protein available for fermentation by gut bacteria in the colon. A reduction in protein digestibility increases the mass of undigested protein entering the colon, thereby enhancing protein fermentation (Zhao et al., 2019). An increase in colonic undigested protein is associated with a significant rise in the abundance of *Clostridium* and *Bacteroidaceae*, as well as increased levels of protein derivatives (*e.g.*, branched-chain amino acids and nitrogen) in the colon and feces (De Brier et al., 2021; Zhao et al., 2019). This metabolic change may regulate emotion-related physiological processes, thereby influencing the risk of mood disorders such as depression (Wang et al., 2023). Concurrently, elevated undigested carbohydrates (*e.g.*, starch and dietary fiber), whose levels are also modulated by extraction rate-dependent changes in endogenous components, promote the production of short-chain fatty acids and inhibit the growth of harmful gut microbiota in the colon, thus promoting host health (Faubel et al., 2025). However, existing studies usually focus on the regulation of gut microbiota by a single macronutrient, such as dietary fiber, overlooking the synergistic interactions between multiple components in wheat flour as modulated by extraction rates, in regulating the gut microenvironment.

Noodles with different wheat flour extraction rates were prepared, and then subjected to *in vitro* static digestion-fermentation experiments. The effects of wheat flour extraction rate on the bioaccessibility of the key components (starch and protein) in noodles, as well as how it regulates the colon microenvironment, were evaluated by analyzing the hydrolysis degree of the starch and protein and the fermentation characteristics of digested residues. This study provides a theoretical foundation for enhancing nutritional precision in wheat processing, minimizing processing losses, and offering a strategy to alleviate the food crisis.

## 2 Materials and methods

### 2.1 Materials

CaCl_2_, NaOH, Na_2_HPO_4_, and NaH_2_PO_4_, *etc.*, were purchased from Xilong Science Co., LTD.; The BCA protein assay kit was acquired from Beijing Solaibao Technology Co., LTD. Pepsin (3100 U/mg), pancreatin (trypsin 139 U/mg; amylase activity 153 U/mg) were purchased from Shanghai Aladdin Biochemical Technology Co., LTD. Total Starch content kit and total dietary fiber kit were procured from Megazyme International Ireland Ltd. The 100 types of online milling streams were obtained from Shandong Luhua (Yanjin) cereal food Co., Ltd. The standard substances of acetic acid, propionic acid, butyric acid, valeric acid and Isovaleric acid were purchased from Shanghai Macklin Biochemical Technology Co., Ltd.

### 2.2 Preparation of noodles

Based on the extraction rates and ash content data of 100 types of online milling streams (Section 2.1), a cumulative ash content-cumulative extraction rate curve was plotted, and wheat flours with 35 %, 75 %, 80 %, 85 %, 90 %, and 95 % cumulative extraction rates were formulated by blending different online milling streams in specific proportions according to this curve. These flours were kneaded with distilled water, with water addition adjusted to 60 % of the optimal water absorption rate determined based on their farinograph curves (Zhang, Qian, et al., 2024). After conditioning at 85 % relative humidity and 30 °C for 30 min, noodles were prepared using a noodle maker. The initial roller gap of the noodle maker was set to 3.0 mm, and the dough was rolled continuously for 4 times. Subsequently, the dough was rolled twice at each of the following roller gaps: 2.2 mm, 1.8 mm, 1.6 mm, 1.4 mm, 1.2 mm, 1.0 mm, and 0.75 mm. Finally, the dough sheet (0.75 mm thick) was cut into noodles with a width of 2.0 mm. After being freeze-dried (freezing temperature: −56 °C, vacuum pressure: 10 Pa, drying time: 40 h), the noodles made from wheat flour with cumulative extraction rates of 35 %, 75 %, 80 %, 85 %, 90 %, and 95 % were designated as M35, M75, M80, M85, M90, and M95, respectively.

### 2.3 *In vitro* gastrointestinal digestion of noodles

The freeze-dried (1.5 g) noodles were crushed into particles <5 mm, mixed with 5 mL of distilled water, and boiled at 100 °C for 6 min. After cooling to 25 °C, the cooked noodles were used for subsequent digestion experiments.

(1) According to Gwala et al. (2020), the effect of salivary amylase on oral digestion could be negligible. Thus, simulated oral digestion was conducted without salivary amylase for 2 min.
(2) Simulated gastric juice was prepared according to INFOGEST *in vitro* static protocol (Brodkorb et al., 2019). Digested chyme was mixed with an equal volume of gastric juice (pH adjusted to 3.0), shaken at 200 rpm for 120 min at 37 °C, and then heated in a boiling water bath for 15 min.
(3) Simulated intestinal fluid was prepared according to INFOGEST static *in vitro* protocol (Brodkorb et al., 2019). The digested chyme was mixed with an equal volume of intestinal fluid (pH adjusted to 7.0). The mixture was oscillated in a water bath at 37 °C with an oscillation rate of 200 rpm. Samples of 0.5 mL were collected at 0, 30, 60, 90, 120, 150, and 180 min during intestinal digestion. After centrifugation at 5440×*g* for 20 min, the supernatant was separated for starch hydrolysis degree determination. Samples collected at 120 min were used to evaluate the protein hydrolysis degree. The precipitates were freeze-dried and stored at 4 °C for subsequent simulated *in vitro* fermentation.

### 2.4 Starch hydrolysis characteristic

#### 2.4.1 Starch hydrolysis degree

The starch hydrolysis degree of intestinal digestion samples was determined using the 3,5-Dinitrosalicylic acid (DNS) colorimetric method. Absorbance was recorded at 540 nm, and a maltose standard curve was used to quantify starch hydrolysis (Wang et al., 2022). The degree of starch hydrolysis was calculated as:

(1) $$\mathrm{Hs}\;\left(\%\right)=0.95\times 100\times M/S_{T}$$

where Hs is the degree of starch hydrolysis, S_T_ is the total starch content in samples, M is the maltose yield produced during intestinal digestion, and 0.95 is the conversion factor of maltose to starch.

#### 2.4.2 Estimated glycemic index

According to Åkerberg et al. (1998), hydrolysis indexes (HI) of noodles with different wheat flour extraction rates were determined by dividing the area under their starch hydrolysis curve by that of the reference sample (fresh white bread). The estimated glycemic index (eGI) was calculated using the following formula:

(2) eGI=8.198+0.862×HI

The starch fractions can be quantified using the following formulas:

(3) $$\mathrm{RDS}\;\left(\%\right)=0.95\times \left(M_{20}–M_{0}\right)/S_{T}$$

(4) $$\mathrm{SDS}\;\left(\%\right)=0.95\times \left(M_{120}-M_{20}\right)/S_{T}$$

(5) $$\mathrm{RS}\;\left(\%\right)=100\%-\mathrm{RDS}–\mathrm{SDS}$$

M_0_ is maltose determined before intestinal digestion; M_20_ and M_120_ are the maltose content determined at 20 min and 120 min of intestinal digestion, respectively. S_T_ is the total starch content in samples.

### 2.5 Protein hydrolysis degree

The analysis of protein hydrolysis degree after 120 min of intestinal digestion was followed (Zhang, Yang, et al., 2024) with minor modifications. A 0.5 mL aliquot of the supernatant was mixed with 0.83 mL of 10 % (wt/vol) trichloroacetic acid, and centrifuged at 6800×*g* for 15 min. The supernatant was combined with 1.5 mL of OPA reagent (0.8 g/mL). The absorbance of the mixture was measured using a microplate reader at 340 nm (Epoch, BioTek, USA). The protein hydrolysis degree (DH) was quantified by the following formula:

(6) $$\mathrm{DH}\left(\%\right)=\frac{h_{s}}{h_{\mathrm{total}}}\times 100\%$$

h_s_ is the moles of free amine groups per gram of protein in the sample, while h_total_ is the moles of total free amino group per gram of protein (7.96 mmol/g protein) (Wen et al., 2020).

### 2.6 *In vitro* colon fermentation

*In vitro* colon fermentation was carried out according to the method of (Zhang, Li, et al., 2024) with slight modifications. The digested residue was heated in a 90 °C water bath for 30 min to sterilize, then cooled to room temperature. Samples (200 mg) were added to a 50 mL anaerobic bottle. Four volunteers (3 men and 1 woman, 25–30 years old, with BMI between 18.5 and 25 kg/m^2^) were selected for this study. These volunteers had no history of intestinal disease, and had not used antibiotics for at least 3 months prior to the study. All fresh fecal were collected from volunteers within 1 h, quickly weighed to 50 g each, and mixed in a sampling bag. The mixed feces were added to autoclaved phosphate buffer (PBS) (0.1 mol/L, pH 7.4, 0.05 % cysteine) at a ratio of 1:5 (m/V), and filtered with a 100-mesh strainer. The fecal filtrate was collected and immediately transferred to an anaerobic incubator for further processing. 16 mL of PBS and 4 mL of fecal filtrate were added to the anaerobic bottle and mixed with the fermentation substrate. A mixture containing only PBS and fecal filtrate served as the blank control. The mixture was incubated under anaerobic conditions (N_2_/H_2_/CO_2_, 90:5:5, *V*/V) at 37 °C. Samples were collected at 0, 4, 8, 12, and 24 h for further analysis. Each sample was analyzed with six repetitions. This study adhered to the World Medical Association Declaration of Helsinki. All participants provided written consent after being fully informed. The study has been approved by the Ethics Committee of Henan University of Technology (Number: HAUT20250312001; date:2025.03.12).

### 2.7 pH and gas production determination

The pH of the fermentation solution was measured using a Micro pH Meter (INESA Scientific Instrument Co., Ltd., Shanghai, China). Before testing, the sensitivity of the Micro pH Meter sensor was calibrated with standard pH buffers of 4.00 and 6.86.

A syringe of 50 mL was inserted into the rubber stopper of the anaerobic bottle, with its internal plunger lubricated with petroleum jelly. Leveraging the syringe's hermeticity and graduated scale, gas generated during *in vitro* fermentation was directly collected, and the gas production is quantified by measuring the volume change within the syringe (Bindelle et al., 2007), with volumes recorded at 0, 4, 8, 12 and 24 h of incubation.

### 2.8 Determination of short-chain fatty acids

The short-chain fatty acids in the *in vitro* fermentation broth prepared in Section 2.6 were analyzed using the method reported by Ferrario et al. (2014), with minor modifications. As follows: After centrifuging at 6770×*g* for 20 min, the supernatant was collected. The supernatant was filtered by a 0.22 μm filter and analyzed using an Agilent 1260 HPLC system (Agilent Technologies Inc., California, USA) with a ZIC-HILIC column (150 × 4.6 mm). The mobile phase was a 7:3 mixture of sodium phosphate buffer solution (15 mmol/L, pH 6.8) and acetonitrile. The flow rate was 0.3 mL/min, the injection volume was 20 μL, and detection wavelength was set at 210 nm. The types and contents of short-chain fatty acids were determined according to the peak time and peak area of acetic acid, propionic acid, butyric acid, valeric acid and 4-methylvaleric acid standards.

### 2.9 Analysis of microbial diversity

The primers used for microbial diversity analysis were 338F (5′-ACTCCTACGGGAGGCAGCAGG-3′) and 806R (5′-GGACTACHVGGGTWT CTAAT-3′). The sequencing platform used in this study was DNBSEQ-G99. Paired-end sequences were spliced using FLASH (v1.2.7) and Pear (v0.9.6) with a minimum overlap of 10 bp and a mismatch rate of 0.1 to obtain the Fasta sequence. 300 bp reads were trimmed at the first base within any 50-bp window with mean Q < 20, then discarded if <50 bp or harboring ambiguous bases. Sequences with 97 % similarity were classified as operational taxonomic units (OTUs). Taxonomic assignment of OTU representatives was performed with RDP Classifier v2.2 against the Silva v138 16S rRNA database at a 70 % confidence cutoff. Chimeric sequences were removed using UCHIME alignment against known databases and DENOVO detection for unknown sequences. Additionally, Short sequences that did not meet the criteria were excluded. Gene function prediction was performed using PICRUSt2 software and analyzed *via* the Greengenes database (http://huttenhower.sph.harvard.edu/galaxy/) (Douglas et al., 2020).

### 2.10 Changes in substrate content during the fermentation

#### 2.10.1 Determination of carbohydrate utilization rate

The determination of carbohydrate utilization rate was performed following described by Gao et al. (2023) with slight adjustments. The fermentation broth prepared in Section 2.6 was centrifuged at 4000×*g* for 15 min at 4 °C. 490 μL of distilled water, 50 μL of centrifuged fermentation liquid, 500 μL of 3 % phenol and 2 mL of concentrated sulfuric acid were mixed in a test tube. After reaction for 30 min, the absorbance value was measured at 490 nm. A standard curve was prepared using a 0.1 mg/mL glucose solution. The carbohydrate utilization was calculated using following equations.

(6) $$\text{Carbohydrate utilization}\left(\%\right)=\frac{C_{0}-C_{T}}{C_{0}}\times 100\%$$

where the C_0_ represents initial glucose concentration after acid hydrolysis, mg/mL; C_T_ represents the glucose concentration after fermentation, mg/mL.

#### 2.10.2 Determination of protein content during the fermentation

The specific method for determining protein content in substrates before and after colonic fermentation was as follows: The precipitate from centrifuged fermentation broth (prepared in Section 2.10.1) was measured using the Kjeldahl method (Bindelle et al., 2007).

(7) $$\text{Protein consumption}\left(\%\right)=\frac{P_{0}-P_{T}}{P_{0}}\times 100\%$$

where the P_0_ represents protein content before fermentation, mg/mL; P_T_ represents the protein content after fermentation, mg/mL.

#### 2.10.3 Determination of free amino acid composition

The method for determining the amino acid concentration in the fermentation broth was referenced from (Zhang, Yang, et al., 2024). 400 μL of the supernatant (prepared in Section 2.10.1) was mixed with 100 μL of 10 % sulfosalicylic acid, and incubated at 4 °C for 60 min. After centrifugation at 6800×*g* for 15 min, the supernatant was diluted 2-fold, passed through a 0.22 μm filter, and then analyzed using an amino acid analyzer (Sykam, Munich, Germany). Each test was performed in triplicate.

(8) $$\text{Amino acid consumption}\left(\%\right)=\frac{A_{0}-A_{T}}{A_{0}}\times 100\%$$

where the A_0_ represents initial amino acid concentration before fermentation, mg/mL; A_T_ represents the amino acid concentration after fermentation, mg/mL.

### 2.11 Statistical analysis

The results were expressed as the mean ± standard deviation of the three repeated trials. IBM SPSS 27 software (SPSS Inc., Chicago, IL, USA) was used to conduct variance (ANOVA) and Duncan test for statistical analysis. *p* < 0.05 indicates that there is statistical significance between the data. α-diversity was evaluated by computing with Chao1 and Simpson using ANOVA. The community composition of each sample was assessed at multiple classification levels (β-diversity) using Principal Component Analysis (PCA). PERMANOVA (permutational multivariate analysis of variance) was used to test the statistical significance of intergroup differences in microbial community structure revealed by PCA. The linear discriminant analysis (LDA) effect size (LEfSe) approach was applied to characterize microbial features (Segata et al., 2011). Using a normalized relative abundance matrix, LEfSe first employs the Kruskal-Wallis rank-sum test to identify features with significant differential abundances among predefined taxa. Subsequently, it uses LDA to evaluate the effect size of each identified feature. In this study, a significance threshold of 0.05 and an LDA score cutoff of 2.0 were used for all biomarker analyses.

## 3 Results and discussion

### 3.1 Starch hydrolysis degree

**Fig. 1:**
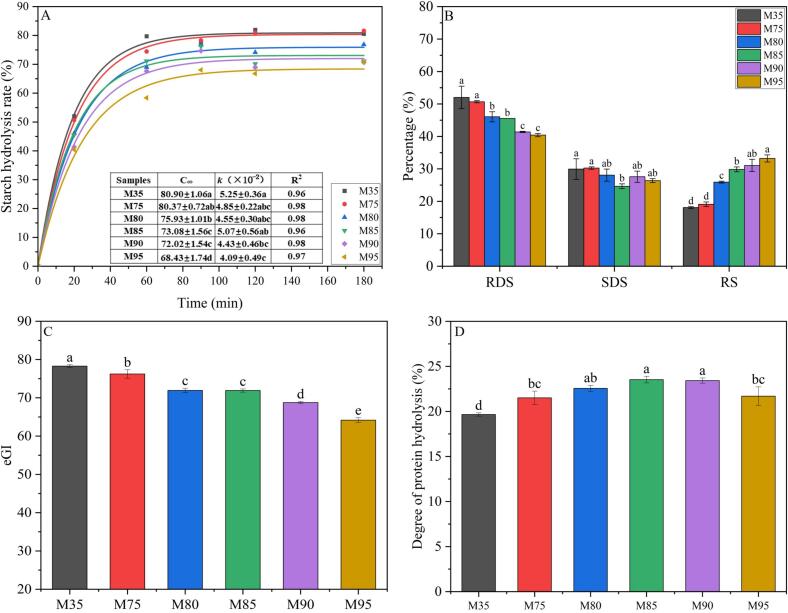
Digestion characteristics of starch and protein in noodles with different wheat flour extraction rates. A: Starch hydrolysis curves; B: Quantitation of rapidly digestible starch (RDS), slow digestible starch (SDS) and resistant starch (RS); C: Estimated glycemic index (eGI); D: Protein hydrolysis degree; M35, M75, M80, M85, M90, and M95 represent noodles with wheat flour extraction rates of 35 %, 75 %, 80 %, 85 %, 90 %, and 95 %, respectively. Different letters in the figure indicate significant differences among the values (*p* < 0.05).

Starch serves as a crucial energy source in wheat flour and its derived products. Evaluating the degree of starch hydrolysis in noodles with different wheat flour extraction rates is of great significance as it can guide consumers with diverse health conditions to maintain a rational diet. Given that starch is primarily hydrolyzed in the small intestine, with minimal hydrolysis occurring in the oral cavity and stomach, the data of starch digestion were collected during the small intestine (Fang et al., 2025). Fig. 1A depicted the starch hydrolysis curves of all noodles during intestinal digestion, indicating that extraction rate significantly influenced the starch digestion curve. The degree of starch hydrolysis increased rapidly in 0–60 min at the beginning of intestinal digestion, and then increased slowly within 60–180 min. The starch hydrolysis data of noodles with different extraction rates were consistent with the first-order model (R^2^ ≥ 0.96). A significant decrease was observed in both the digestion rate constant (*k*) and C_∞_, indicating that the wheat flour extraction rate significantly reduced both the digestion rate and the amount of digestible starch in the noodles. Notably, the proportion of resistant starch (RS) increased from 18.07 % to 33.23 % as extraction rate rose from 35 % to 95 % (Fig. 1B). RS is not digested in the small intestine but fermented by gut microbiota in the colon, producing short-chain fatty acids (SCFAs) such as butyrate—metabolites proven to enhance intestinal barrier function, reduce systemic inflammation and type 2 diabetes (Venkataraman et al., 2016). This aligns with the observed reduction in starch hydrolysis, as RS contributes to the lower C_∞_ values in high-extraction-rate noodles. This reduction was likely due to an enhanced physical shielding effect resulting from the elevated levels of protein and wheat bran dietary fiber in noodles with higher flour extraction rates (Zhang, Qian, et al., 2024). Additionally, the inhibitory effect of dietary fiber on amylase activity may further hinder starch digestion (Zhu et al., 2022).

To translate these kinetic changes into health relevance, we analyzed the estimated glycemic index (eGI) (Fig. 1C), which is a validated proxy for postprandial blood glucose responses (Ji et al., 2024). The eGI reflects only the effect of carbohydrates on blood sugar levels, not the amount of carbohydrates. According to the classification of carbohydrate foods, low eGI is defined as ≤55, medium eGI is defined as >55 to <70, and high eGI is defined as ≥70. Noodles with a wheat flour extraction rate below 90 % were classified as high eGI, while those with 90 % and 95 % wheat flour extraction rate were categorized as medium eGI. This classification suggests that increasing extraction rate may lower the eGI of noodles, potentially due to higher levels of protein and dietary fiber, which can slow carbohydrate digestion and absorption. However, this decrease in starch digestibility in noodles with high extraction rates (≥90 %) may also lead to a reduction in net energy intake, which needs to be weighed carefully in economically underdeveloped regions where staple foods constitute the primary energy source. Differentiated application strategies are recommended based on the specific needs of individuals. Noodles with high flour extraction rates (≥90 %) are particularly recommended for individuals at high risk of diabetes or overnutrition, while in areas with insufficient caloric intake, noodles with lower extraction rates can be preferred.

### 3.2 Protein hydrolysis degree

The degree of protein hydrolysis of noodles with different wheat flour extraction rates was shown in Fig. 1D. The protein hydrolysis degree of noodles at the end of the intestinal stage was significantly affected by extraction rate. The protein hydrolysis degree after gastrointestinal digestion increased from 19.66 % (M35) to 23.53 % (M85), with M80, M85 and M90 exhibiting no significant difference but being notably higher than other samples. This indicated that increasing from 35 % to 80 %–90 % enhanced protein utilization in wheat flour. This was consistent with our previous studies (Zhang, Qian, et al., 2024; Zhang, Yang, et al., 2024), which found that a moderate increase in extraction rate (80 %–85 %) could promote an increase in protein digestibility and an increase in the release of amino acids in porridge made from wheat flour. This may be attributed to the higher proportion of albumin and globulin from the aleurone layer in wheat flour with a flour extraction rate of ≥80 % (Zhang, Yang, et al., 2024). It was reported that the digestibility of albumin and globulin was significantly higher than that of gluten protein in wheat flour (Ingrid et al., 2025). Therefore, the increased proportion of albumin and globulin significantly improved the hydrolysis degree of protein in wheat flour of 80 %–90 % extraction rates (Supplemental Table 1). Elevated hydrolysis degree of protein in this range translates to greater availability of essential amino acids (*e.g.*, lysine, methionine)—nutrients critical for muscle protein synthesis and immune function (Fang et al., 2025; Li et al., 2023). With the further increase of the flour extraction rate, the digestibility of protein has decreased to 21.69 % in M95, likely due to excessive wheat bran fiber in the cortex, consistent with previous findings that high fiber content can hinder protein hydrolysis (Zhang, Qian, et al., 2024). Therefore, increasing the flour extraction rate of wheat flour to 80 %–90 % can enhance its protein nutritional value.

### 3.3 Changes in pH during *in vitro* colon fermentation

**Fig. 2:**
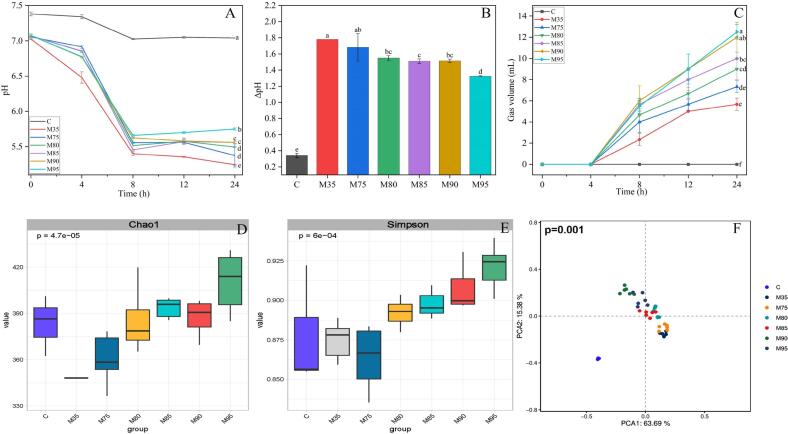
Fermentation characteristics of noodles with different wheat flour extraction rates. A: pH change; B: ΔpH; C: Gas production volume; D: α-diversity (Chao1 index); E: α-diversity (Simpson index); F: β-diversity (Principal component analysis, PCA); M35, M75, M80, M85, M90, and M95 represent noodles with wheat flour extraction rates of 35 %, 75 %, 80 %, 85 %, 90 %, and 95 %, respectively. Different letters in the A–F indicate significant differences among the values (p < 0.05) as determined by ANOVA; Group separation in PCA with was confirmed significant by PERMANOVA (*p* = 0.001).

During *in vitro* fermentation, the pH of fermentation solution decreases with the prolonged fermentation, with the accumulation of short-chain fatty acids (SCFAs) by gut microbiota. As presented in Fig. 2A and B, the pH of the control group (group C) remained relatively stable (7.04–7.38), due to the absence of substrates carbohydrates and proteins. In contrast, the pH of all noodles decreased significantly within the first 8 h, a trend consistent with previous studies. This was due to the easy fermentability of resistant starch and soluble dietary fiber, resulting in the rapid production of SCFAs (Fan et al., 2025). After 8 h of fermentation, the pH decline slowed across all noodles. Notably, throughout the fermentation process, the pH of noodles with a 35 % flour extraction rate (M35) exhibited a continuous decline. The pH of the other samples displayed a slight increase following 12 h of fermentation. This phenomenon may be because the increased content of insoluble wheat bran dietary fiber in noodles increased, which slowed the fermentation rate and reduced the production rate of SCFAs. Moreover, a higher level of undigested protein in the noodles with 90 % and 95 % extraction rate, which promoted protein fermentation and the generation of alkaline metabolites, causing pH to rise again (Feng et al., 2022). Notably, pH curves differed significantly between groups: noodles with higher extraction rates had significantly higher pH than those with lower rates. Previous research reported a similar trend, with (De Brier et al., 2021) finding that wheat flour with a higher wheat bran content had a significantly higher pH than wheat flour with less wheat bran content.

### 3.4 Gas production during *in vitro* colon fermentation

The production of gas, including H_2_, CH_4_, and CO_2_, serves as an indicator of gut microbiota fermentation activity, which can be enhanced by a high level of dietary fiber. Significant differences in gas production were observed among noodles with varying wheat flour extraction rates during fermentation (Fig. 2C). Notably, the control group exhibited no gas production throughout the fermentation process due to the absence of fermentable substrates. Unlike the control group, all samples demonstrated gas production after 4 h of fermentation, with gas volume positively correlated with flour extraction rate. After 24 h of fermentation, M90 (12 mL) and M95 (12.5 mL) exhibited markedly higher gas production compared to other samples, potentially due to their higher residual protein content and greater insoluble dietary fiber levels. This observation aligned with prior studies demonstrating that insoluble dietary fiber promotes more substantial gas production compared to soluble dietary fiber alone, which may reduce the host's intestinal comfort.

### 3.5 Analysis of gut microbiota diversity after fermentation

The variation of wheat flour extraction rate alters the composition and structure of protein and starch in noodles, thus modulating microbial metabolism and gut microbiota diversity. The diversity of gut microbiota plays an important role in maintaining the normal operation of the host's energy metabolism and immune regulation (Wu et al., 2021). 16S rDNA sequencing was conducted to characterize the gut microbiota after *in vitro* fermentation. Species richness (Chao1 index) was significantly higher in groups fermented with noodles of varying extraction rates (Fig. 2D and E). Moreover, the Simpson index, which assessed evenness, showed a significant increase in the gut microbiota associated with higher wheat flour extraction rates (*p* < 0.05). Beta-diversity (differences in microbial composition between samples) was analyzed *via* principal component analysis (PCA) based on OTU relative abundances (Fig. 2F). The first two principal components (PC1 and PC2) explained 63.69 % and 15.38 % of the variance, respectively. Noodles with high extraction rates (M90 and M95) showed significant separation from the other samples, indicating noteworthy differences in their gut microbiota (*p* = 0.001, PERMANOVA). In contrast, M35 and M75 exhibited partial overlap in their microbial profiles, suggesting comparable community structures at noodles with ≤75 % extraction rates. These results revealed a critical threshold effect: noodles with extraction rates >75 % induced significant shifts in gut microbiota, whereas those with ≤75 % had negligible impacts.

### 3.6 Changes of gut microbiota abundance at phylum level and genus level

**Fig. 3:**
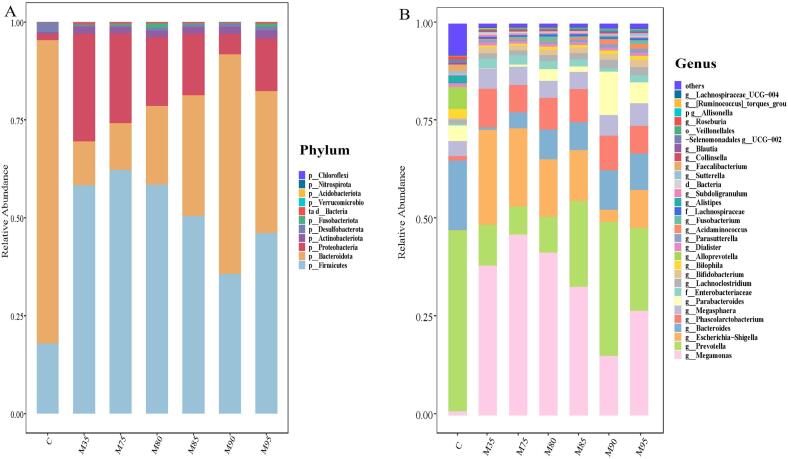
Relative abundances at the phylum and genus levels of gut microbiota after 24 h *in vitro* colon fermentation. A: Relative abundances at the phylum; B: Relative abundances at the genus; M35, M75, M80, M85, M90, and M95 represent noodles with wheat flour extraction rates of 35 %, 75 %, 80 %, 85 %, 90 %, and 95 %, respectively.

The gut microbiota composition of noodles with different extraction rates at the phylum level after *in vitro* fermentation was shown in Fig. 3A. After fermentation for 24 h, Firmicutes, Bacteroidetes, Proteobacteria and Actinobacteria were the main components of gut microbiota at the phylum level. Increasing flour extraction rate was associated with a significant reduction in Firmicutes abundance (from 58.23 % to 35.64 %) and a concurrent increase in Bacteroidetes abundance (from 11.22 % to 56.05 %), leading to a marked elevation in the Bacteroidetes/Firmicutes ratio. An elevated Bacteroidetes/Firmicutes ratio is linked to reduced insulin resistance and weight gain risk (Wu et al., 2021), suggesting higher-extraction noodles may exert anti-obesity effects *via* this regulatory pathway. In addition, the abundance of Firmicutes was positively correlated with the degree of protein fermentation (Zhang, Li, et al., 2024), which means that the increase in wheat flour extraction rate is conducive to reducing the protein fermentation of noodles in the colon.

The abundance of Proteobacteria in noodles was negatively correlated with extraction rate. Among Proteobacteria, several pathogenic bacteria such as *Shigella*, *Escherichia*, and *Salmonella* are known inducers of intestinal inflammation, which are harmful to host health (Faubel et al., 2025). Elevated Proteobacteria levels are widely recognized as biomarkers of microbial dysbiosis (Gomez-Gomez et al., 2025). Compared with noodles with low extraction rates (≤75 %), the abundance in Proteobacteria of noodles with high extraction rates (≥90 %) was significantly reduced, suggesting that noodles with high flour extraction rate may inhibit the proliferation of Proteobacteria in the colon and potentially lower the risk of intestinal inflammatory diseases. Conversely, the abundance of Actinobacteria gradually increased with the increase of flour extraction rate, suggesting that wheat flour with high extraction rates could promote the proliferation of actinomycetes. Bifidobacterium, a prominent probiotic within the phylum Actinobacteria, helps control serum cholesterol levels, prevents intestinal diseases, regulates the immune system, and exhibits anti-cancer activity (Diana et al., 2014). The stimulation of Actinobacteria growth assumed to the high content of wheat bran dietary fiber in noodles of high wheat flour extraction rates, especially arabinoxylan, resistant starch and β-glucan, which could promote the proliferation of Actinobacteria (Faubel et al., 2025).

At the genus level (Fig. 3B), as the extraction rate increased, the abundance of *Megamonas* exhibited a trend of first increasing and then decreasing, reaching the lowest value in M90. Compared with the M35 group, the abundance of *Megamonas* in M90 decreased by 23.00 %. *Megamonas* is involved in the fermentation of various carbohydrates, producing acetic, propionic, and lactic acid as end products. A reduced abundance of *Megamonas* was associated with a lower risk of obesity and metabolic dysfunction. *Prevotella* exhibited the second highest abundance, surpassing *Bacteroides*. This distribution pattern was consistent with the gut microbiota profiles typically observed in Asian populations, where *Prevotella* dominance correlates with dietary habits rich in complex carbohydrates (Precup & Vodnar, 2019). The abundances of *Prevotella* and *Bacteroides* in noodles showed an increasing trend with the increase in extraction rate. *Prevotella* abundance increased with extraction rate, likely because of two factors: (1) higher levels of resistant starch and insoluble wheat-bran fiber (Zhang, Yang, et al., 2024), and (2) reduced protein digestibility, which has been reported to promote *Prevotella* growth (Zhang, Li, et al., 2024). *Bacteroides*, which ferments of resistant starch and soluble dietary fiber, plays an important role in improving the metabolism and immune disorders of obesity (Fan et al., 2025). The relative abundance of *Bacteroides* increased with the extraction rate, which can be attributed to the increase of undigested starch and soluble dietary fiber in wheat flour (Akihito et al., 2020). It was worth noting that the abundance of *Escherichia-Shigella* in Proteobacteria, a potential pro-inflammatory bacterium, was significantly reduced in high-flour-extraction rate wheat noodles and reached its lowest value in M90 (De Brier et al., 2021). The intestinal core bacteria *Parabacteroides* increased with flour extraction rate and was higher in diets rich in resistant starch. A similar trend was observed for *g__Lachnoclostridium*, suggesting enhanced anti-inflammatory effects with increased flour extraction rate (Cui et al., 2022). Furthermore, *Fusobacterium* levels were reduced in M90 and M95, which has been linked to a lower risk of gastric tumors.

While our study used a pooled fecal inoculum without separate analysis of individual donor data, we acknowledge that interindividual gut microbiota variability is a key consideration, which is driven by diet, genetics, and lifestyle (Yang & Rose, 2014). This variability could lead to individual-specific responses to wheat extraction rates, which our design does not capture. Notably, our key findings (*e.g.*, elevated gut microbiota diversity) align with previous reports on nutrients fermentation (Zhang, Li, et al., 2024), supporting the validity of observed trends. Future studies with larger cohorts and individual-level analyses will be critical to quantify how interindividual microbial differences modulate these effects—for example, whether specific enterotypes are more responsive to extraction rate changes.

### 3.7 Linear discriminant analysis of gut microbiota

**Fig. 4:**
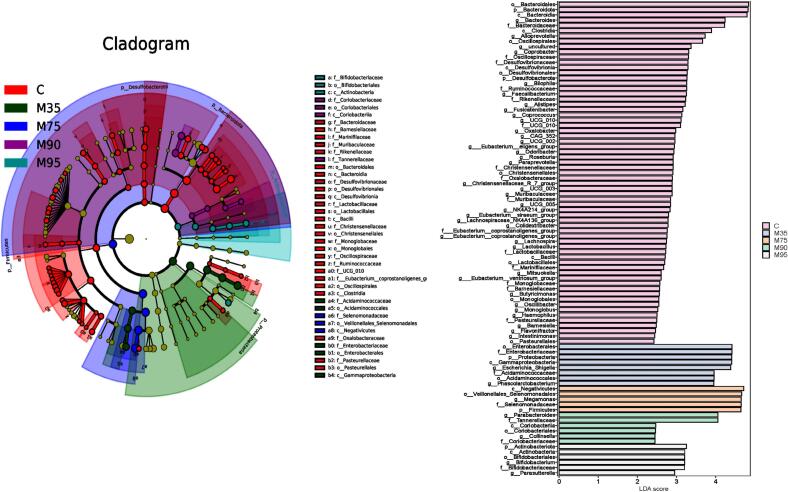
Linear discriminant analysis of gut microbiota after 24 h *in vitro* colon fermentation; M35, M75, M80, M85, M90, and M95 represent noodles made from wheat flour with extraction rates of 35 %, 75 %, 80 %, 85 %, 90 %, and 95 %, respectively. The analysis was performed with a screening threshold of LDA score > 2.0 and *p* < 0.05.

According to the linear discriminant analysis (LDA), the characteristic gut microbiota of noodles with different wheat flour extraction rate was evaluated (Fig. 4). At the phylum level, Proteobacteria and Firmicutes were enriched in the M35 (LDA > 2), while Firmicutes were enriched in the M75 (LDA > 2). Bacteroidetes and Actinobacteria (LDA > 2) were enriched in M90, and Actinobacteria (LDA > 2) were enriched in the M95, particularly with a notable enrichment of *Bifidobacterium*. No significant enrichment was observed at the phylum level in the M80 and M85, suggesting a higher degree of stability of their gut microbiota. This stability contributes to maintaining the gut microbiota balance, reducing disease susceptibility, and supporting host health. Noodles with an extraction rate of 80 %–85 % exhibited a higher potential for promoting the stability of gut microbiota, whereas noodles with a wheat flour extraction rate of 90 %–95 % showed a greater tendency for targeted modulation of specific gut microbiota, such as *Bifidobacterium*.

### 3.8 Production of major short-chain fatty acids

**Fig. 5:**
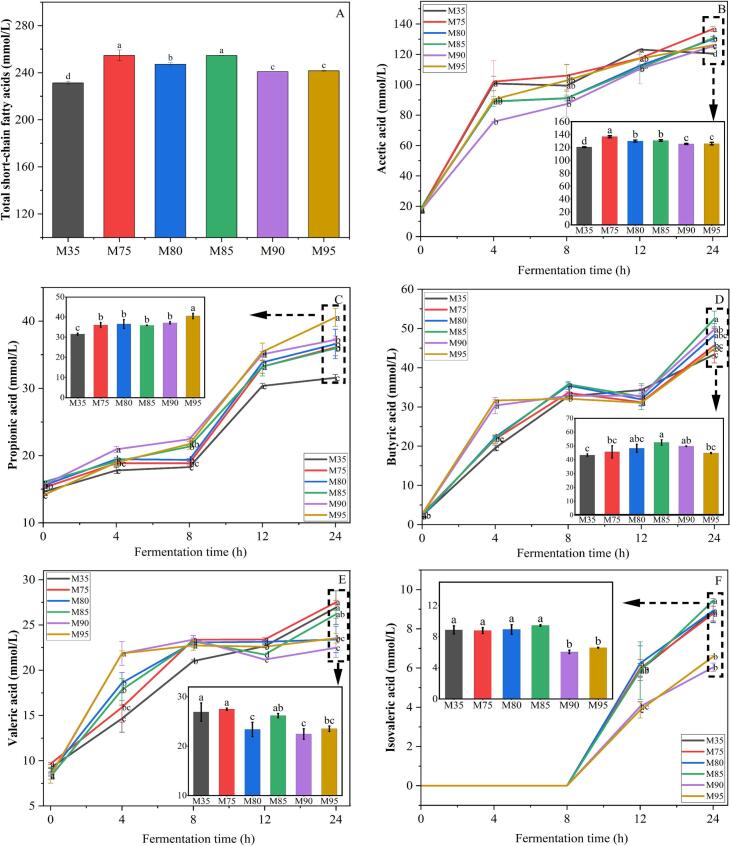
Production of major short-chain fatty acids during 24 h *in vitro* colon fermentation. A: Total SCFAs; B: Acetic acid; C: Propionic acid; D: Butyric acid; E: Valeric acid; F: Isovaleric acid; M35, M75, M80, M85, M90, and M95 represent noodles made from wheat flour with extraction rates of 35 %, 75 %, 80 %, 85 %, 90 %, and 95 %, respectively. Different letters in the figure indicate significant differences among the values (*p* < 0.05).

Short-chain fatty acids (SCFAs), mainly including acetic acid, propionic acid, butyric acid, and valeric acid, are primarily produced during the fermentation of undigested substrates such as protein, starch, and dietary fiber by gut microbiota in the colon (Yang & Rose, 2014). SCFAs play a crucial role in regulating energy balance, modulating intestinal inflammation signaling pathways, and enhancing insulin sensitivity. Notably, changes in dietary nutrient components can significantly influence the composition and concentration of SCFAs. The results showed that the yield of SCFAs was significantly affected by the wheat flour extraction rate (Fig. 5A). Compared with the 35 % extraction rate group, higher extraction rates significantly increased SCFAs production. Specifically, noodles with 75 %–85 % extraction rates exhibited the highest SCFAs levels, reaching 247.19–254.73 mmol/L, which were significantly higher than those in other samples.

Acetic acid, propionic acid, butyric acid, and valeric acid contents during fermentation are presented in Fig. 5B–E. Acetic acid was found to be the most abundant SCFA after the colon fermentation, aligning with previous studies. Acetic acid increased rapidly within the first 4 h, then slowed but continued to rise. After 24 h of fermentation, wheat noodle samples with a flour extraction rate of 75 %–85 % exhibited significantly higher acetic acid content (129.13–136.67 mmol/L) compared to other samples. This may be attributed to the higher residual levels of starch and soluble dietary fiber in noodles with high extraction rates after small intestine digestion, as acetic acid is a primary product of starch and soluble dietary fiber fermentation *in vitro*. However, the elevated content of less fermentable insoluble dietary fiber, in high-flour-extraction rate wheat noodles may reduce acetic acid content. Therefore, the output of acetic acid decreased slightly in noodles with a higher flour extraction rate. Previous research has demonstrated that elevated acetic acid levels can suppress appetite by acting on central homeostatic mechanisms across the blood-brain barrier (Fang et al., 2025). Propionic acid levels rose rapidly within the first 12 h of fermentation, consistent with the rapid fermentation of resistant starch and soluble dietary fiber in the proximal colon (Fig. 5C). After 24 h of fermentation, the content of propionic acid gradually increased with the flour extraction rate and reached a maximum of 40.56 % in the M95. Propionic acid production correlates with insoluble dietary fiber (Fang et al., 2025), explaining why M95 (highest insoluble bran fiber) had the highest levels. Propionic acid reduces serum cholesterol, prevent diet-induced obesity, and improve insulin sensitivity (Wu et al., 2021), suggesting higher extraction rates may enhance anti-obesity effects.

Butyric acid, the primary energy source for colon epithelial cells, alleviates insulin resistance and diabetes and regulates host gene expression, cell differentiation, and apoptosis (Fang et al., 2025). As shown in Fig. 5D, butyric acid production also increased rapidly within the first 4 h. The M90 and M95 exhibited a significantly higher increase rate of butyric acid due to their higher content of resistant starch and soluble dietary fiber. After 24 h of fermentation, butyric acid content showed significant variation across different flour extraction rates. Specifically, the M35 had the lowest butyric acid content at 43.39 mmol/L. Butyric acid content gradually increased to a maximum of 52.45 mmol/L in the M85 and then decreased with further increases in flour extraction rate. The content of valeric acid is negatively correlated with the severity of ulcerative colitis (Liu et al., 2024). As shown in Fig. 5E, the valeric acid content in M35 and M75 exhibited higher values than that in other samples. This indicated that noodles with a flour extraction rate ≥ 80 % may not be beneficial for patients with ulcerative colitis.

Isovaleric acid (a branched-chain fatty acid, BCFA) is a marker of bacterial protein fermentation in the colon (low concentration) and is derived from amino acid fermentation (valine, isoleucine, leucine) (De Brier et al., 2021). As depicted in Fig. 5F, the production of BCFAs began after 12 h of fermentation, aligning with findings reported by (De Brier et al., 2021), who reported that carbohydrate fermentation predominates in the early stages of fermentation, while protein fermentation is primarily concentrated in later stage of fermentation. After 24 h of fermentation, high-flour-extraction rate wheat noodles (M90 and M95) exhibited significantly lower BCFAs yields compared to other samples, indicating their protein fermentation was inhibited. This may be attributed to the higher content of undigested starch and dietary fiber in high-flour-extraction rate wheat noodles (Supplemental Table 1), which may preferentially utilize carbohydrates as substrates, thereby reducing protein fermentation.

### 3.9 Changes in substrate content during the fermentation

#### 3.9.1 Change in carbohydrate utilization rate

**Fig. 6:**
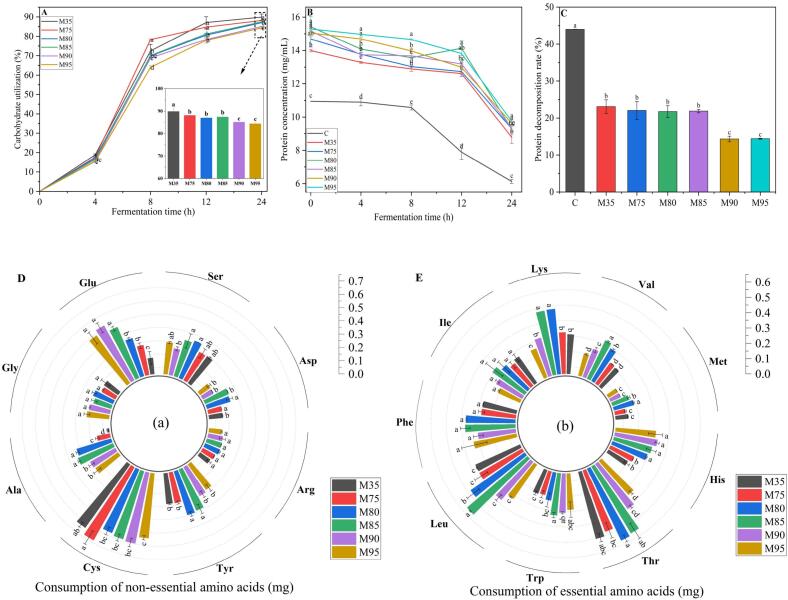
The consumption of substrates during 24 h *in vitro* colon fermentation. A: The consumption of protein； B: The consumption of carbohydrates; C: The consumption of non-essential amino acids; D: The consumption of essential amino acids; M35, M75, M80, M85, M90, and M95 represent noodles with wheat flour extraction rates of 35 %, 75 %, 80 %, 85 %, 90 %, and 95 %, respectively. Different letters in the figure indicate significant differences among the values (*p* < 0.05).

Evaluating the carbohydrate utilization rate of noodles with different wheat flour extraction rates is helpful for better understanding its effects on gut health during the colon fermentation. Therefore, the changes in carbohydrate utilization rate during the fermentation of noodles with different wheat flour extraction rates were analyzed. As shown in Fig. 6A, in all the samples, the carbohydrate utilization rate increased rapidly during the first 8 h of fermentation. Among them, the carbohydrate utilization rate in M35 and M75 increased significantly faster than that in other samples. This was because the gastrointestinal digestion residues in noodles with 35 % and 75 % extraction rates were primarily composed of undigested starch. Undigested starch, due to its highly fermentable nature, can be quickly utilized by intestinal microbiota. In contrast, noodles with an extraction rate exceeding 80 % contained a relatively high proportion of insoluble wheat bran dietary fiber (Supplemental Table 1). The low fermentability of this insoluble wheat bran dietary fiber reduced carbohydrate utilization in the M90 and M95. Overall, the extraction rate of noodles was significantly negatively correlated with the carbohydrate utilization rate.

#### 3.9.2 Changes in protein content during fermentation

The fermentation of proteins in the colon is generally considered to have a dual effect on gut health. On the one hand, the amines produced by protein fermentation help to buffer host stress and reduce the incidence of depression (Portune et al., 2016). On the other hand, the extensive fermentation of protein can produce a series of toxic fermentation products such as amines, indoles, phenolic compounds, and amines (De Brier et al., 2021). Therefore, further analysis of the change in protein consumption during the fermentation is valuable for aiding consumers in selecting optimal wheat flour for their requirements. As fermentation time increased, the protein contents in the fermentation solution of noodles with different wheat flour extraction rates decreased, with a rapid reduction observed after 12 h of fermentation. (Fig. 6B). This finding could be attributed to the preferential utilization of resistant starch and soluble dietary fiber by gut microbiota. During the initial 12 h of fermentation, the abundance of fermentable carbohydrates inhibited the fermentation of proteins. Once the fermentation time exceeded 12 h, the contents of resistant starch and soluble dietary fiber declined, and consequently, the fermentation rate of protein was enhanced, thus resulting in a prominent decline in protein concentration. Previous studies have also confirmed that, due to the extensive fermentation of carbohydrates in the proximal colon, the availability of carbohydrates in the distal colon gradually decreased, resulting in a higher degree of protein fermentation (De Brier et al., 2021).

As illustrated in Fig. 6B and C, after fermentation for 24 h, the protein consumption rates of noodles with 90 % and 95 % extraction rate were significantly lower than that of other noodles. This may be due to the reduced abundance of Proteobacteria, particularly *Escherichia-Shigella*, which is closely associated with protein fermentation (Faubel et al., 2025). In addition, the abundance of resistant starch and dietary fiber in M90 and M95, which serve as the primary substrates for gut microbiota, led to a decreased demand for protein by gut microbiota, thereby reducing protein consumption. This conclusion was supported by the findings of Faubel et al. (2025), who demonstrated that the presence of dietary fiber reduced the fermentation of proteins and amino acids, thereby decreasing the formation of nitrogen compounds such as ammonia. Therefore, noodles with a flour extraction rate of 80 %–85 % were suitable for consumers who want more protein nutritional value. Noodles with 90 % and 95 % flour extraction rate exhibited lower protein consumption rates during colon fermentation and were suitable for consumers who already consumed adequate dietary protein, helping to mitigate the health risks associated with excessive protein intake.

#### 3.9.3 Amino acid consumption

Gut microbiota can regulate host bioavailability of free amino acids through utilization of free amino acids in the colon (Li et al., 2024). Therefore, analyzing amino acid consumption in wheat noodles with varying flour extraction rates within the colonic environment is essential for guiding consumers toward a balanced diet. As shown in Fig. 6C, the amino acid consumption of noodles with different flour extraction rates after 24 h of *in vitro* fermentation. The consumption of amino acids by gut microbiota was significantly affected by the wheat flour extraction rate. Total amino acid consumption, essential amino acid consumption, and non-essential amino acid consumption all exhibited a similar trend of initially increasing and then decreasing, reaching maximum values at 85 % extraction rate of 5.25 mg, 2.97 mg, and 2.32 mg, respectively. The decrease in amino acid consumption in noodles with 90 % and 95 % flour extraction rate may be related to the decrease in the relative abundance of gut microbiota associated with amino acid metabolism induced by high level of dietary fiber (Supplemental Fig. 1). A key limitation for industrial scalability is that the higher extraction rate (≥80 %) retains more bran and germ, potentially compromising dough machinability (*e.g.*, reduced elasticity) and noodle texture (*e.g.*, darker color), which may affect consumer acceptance. Future studies could extend these investigations to fermented products such as steamed buns, evaluating whether alternative processing approaches can alleviate bran-induced textural impairments while preserving nutritional attributes.

## 4 Conclusion

This study revealed that the wheat flour extraction rate regulated the digestive characteristics of major nutrients (starch and protein) and gut microbiota by altering the contents of endogenous nutrients in noodles. Specifically, the degree of starch hydrolysis in noodles significantly decreased with the increased wheat flour extraction rate, reaching the lowest value in noodles with a flour extraction rate of 95 %, while the degree of protein hydrolysis in noodles with flour extraction rates of 80 %–90 % was higher than that of other samples. These changes also modified the colon fermentation characteristics of noodles with different wheat flour extraction rates. After fermentation, the increased wheat flour extraction rate promoted the diversity and uniformity of the gut microbiota. The ratio of Bacteroidetes/Firmicutes increased with the wheat flour extraction rate, positively modulating the gut microbiota. Notably, when the wheat flour extraction rate increased to 80 %–85 %, no specific gut microbiota enrichment was observed in noodles, highlighting their ability to maintain the balance of gut microecology. Noodles with a higher extraction rate (≥90 %) showed the enrichment of Actinobacteria, suggesting the ability of targeted gut microbiota regulation (*e.g.*, *Bifidobacterium)*. Moreover, noodles with a wheat flour extraction rate of 80 %–85 % produced more SCFAs and less gas. In summary, wheat noodles with a flour extraction rate of 80 % to 85 % were suitable for consumers seeking enhanced protein nutrition, such as fitness enthusiasts and vegetarians. Noodles with a wheat flour extraction rate of 90 %–95 % showed lower starch and protein hydrolysis degrees in the small intestine and reduced protein fermentation degrees in the colon, making them ideal for those with high dietary protein intake to mitigate health risks. This study provided theoretical support for wheat flour mills to develop “nutritious” (80 %–85 %) and “health-regulating” (90 %–95 %) specialized flour in a targeted manner by optimizing the flour extraction rate, thereby offering a targeted strategy to contribute to more efficient resource utilization and enhanced health benefits. A key limitation for industrial scalability is that wheat flour with higher extraction rates (≥80 %) retains more bran and germ, which may increase equipment wear (due to bran hardness) and reduce production efficiency (*e.g.*, decreased flour flowability) during milling. Additionally, germ-derived fats raise concerns about storage stability (*e.g.*, accelerated oxidation and rancidity). Future perspectives could focus on: (1) optimizing milling technologies (*e.g.*, modified roller configurations) to mitigate equipment wear while retaining nutrients; (2) exploring natural antioxidants to enhance storage stability of high-extraction flour.

## CRediT authorship contribution statement

**Lingfang Zhang:** Writing – review & editing, Writing – original draft, Validation, Software, Methodology, Investigation, Data curation, Conceptualization. **Peng Chen:** Validation, Software, Investigation, Data curation. **Haowen Lu:** Validation, Software, Investigation. **Qianyi Jiang:** Validation, Software, Data curation. **Fan Yang:** Investigation, Data curation. **Binghua Sun:** Writing – review & editing, Supervision, Conceptualization. **Xiaoxi Wang:** Supervision, Resources, Project administration, Funding acquisition.

## Ethical guidelines

This study adhered to the World Medical Association Declaration of Helsinki. All participants provided written consent after being fully informed. The study has been approved by the Ethics Committee of Henan University of Technology (number: HAUT20250312001; date:2025.03.12).

## Declaration of competing interest

The authors declare that they have no known competing financial interests or personal relationships that could have appeared to influence the work reported in this paper.

## Data Availability

Data will be made available on request.
