# Supplementary material for: Increasing wheat flour extraction rate for balanced starch-protein digestion and gut microbiota optimization: A strategy to enhance nutrition and mitigate food crises
Source: Food Chem X. 2025 Jul 30;29:102848. doi: 10.1016/j.fochx.2025.102848 (PMC12337690; doi:10.1016/j.fochx.2025.102848)
Supplement: Supplementary material [file mmc1.docx]

Supplemental Table 1 The components of noodles with different wheat flour extraction rates (% of sample dry mass)

| Flour extraction rate  (%) | Protein content (%) | Starch content (%) | Insoluble dietary fiber content (%) | | Soluble dietary fiber content (%) | Total dietary fiber content (%) | Relative content (% of total protein) | | | |
| --- | --- | --- | --- | --- | --- | --- | --- | --- | --- | --- |
|  |  |  |  |  |  |  | Albumin | Globulin | Gliadin | Glutenin |
| 35 | 11.28±0.22^d^ | 84.23±0.46^a^ | | 2.10±0.04^f^ | 1.94±0.26^c^ | 4.04±0.22f | 6.00±0.48^d^ | 5.13±0.45^c^ | 5.13±0.45^c^ | 45.99±1.21^a^ |
| 75 | 13.19±0.08^c^ | 80.28±0.07^b^ | | 3.70±0.20^e^ | 2.11±0.20^bc^ | 5.81±0.01e | 6.45±0.17^cd^ | 6.77±0.04^b^ | 6.77±0.04^b^ | 38.07±0.99^b^ |
| 80 | 13.40±0.08^c^ | 78.53±0.27^c^ | | 5.00±0.06^d^ | 2.19±0.16^bc^ | 7.20±0.23d | 6.36±0.49^bc^ | 7.56±0.13^a^ | 7.56±0.13^a^ | 34.95±1.56^c^ |
| 85 | 13.78±0.48^bc^ | 75.90±0.94^d^ | | 6.72±0.23^c^ | 2.54±0.19^ab^ | 9.26±0.42c | 6.90±0.35^bc^ | 7.60±0.06^a^ | 7.60±0.06^a^ | 29.22±0.78^d^ |
| 90 | 14.06±0.28^b^ | 72.63±0.46^e^ | | 9.40±0.24^b^ | 2.56±0.11^ab^ | 11.96±0.13b | 7.29±0.16^ab^ | 7.35±0.16^ab^ | 7.35±0.16^ab^ | 27.06±2.01^d^ |
| 95 | 14.66±0.03^a^ | 69.48±0.23^f^ | | 11.32±0.21^a^ | 2.83±0.06^a^ | 14.15±0.27a | 7.61±0.11^ab^ | 7.74±0.32^a^ | 7.74±0.32^a^ | 22.65±1.34^e^ |

Different superscript letters in the same column indicate significant differences among the values (*p* < 0.05).

Supplemental Table 2 Standard curves of five short-chain fatty acids

| Types | Standard curve | R^2^ |
| --- | --- | --- |
| Acetic acid | y=8.54x-3.09 | 0.9988 |
| Propionic acid | y=15.13x-556.86 | 0.9940 |
| Butyric acid | y=8.49x-1.49 | 0.9997 |
| Valeric acid | y=12.85x-1.09 | 0.9998 |
| Isovaleric acid | y=10.59x+1.02 | 0.9997 |

Supplemental Fig.1


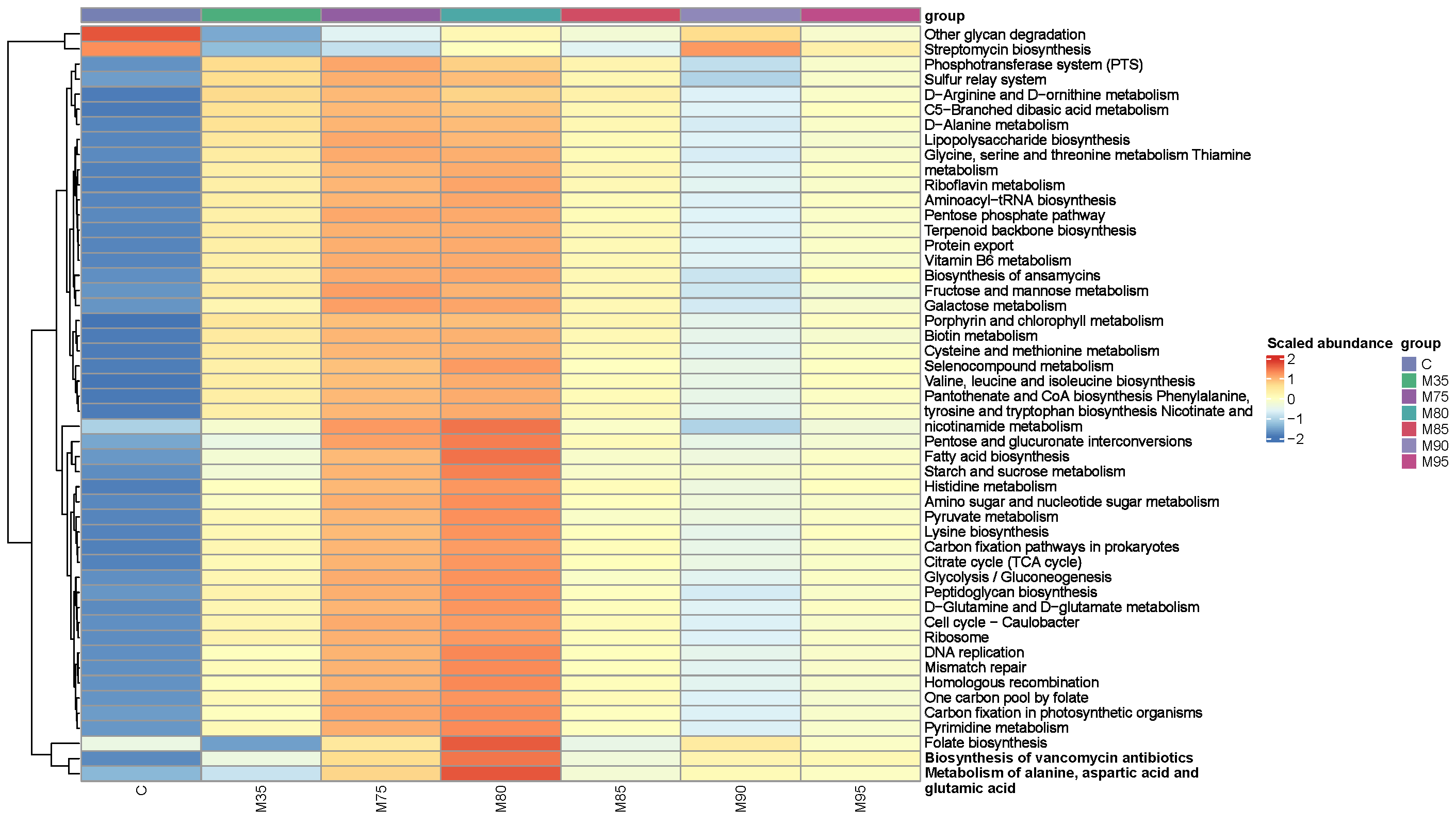


Supplemental Fig.1 Functional changes of gut microbiota in noodles with different wheat flour extraction rates after 24 h *in vitro* fermentation.
